# Investigation of structural and reflective characteristics of short-period Mo/B_4_C multilayer X-ray mirrors

**DOI:** 10.1107/S1600577524000419

**Published:** 2024-02-09

**Authors:** Roman Shaposhnikov, Vladimir Polkovnikov, Sergey Garakhin, Yuliy Vainer, Nikolay Chkhalo, Ruslan Smertin, Kirill Durov, Egor Glushkov, Sergey Yakunin, Mikhail Borisov

**Affiliations:** a The Institute for Physics of Microstructures of the Russian Academy of Sciences, Academicheskaya, Nizhny Novgorod 603087, Russian Federation; b National Research Center ‘Kurchatov Institute’, Kurchatov, Moscow 123182, Russian Federation; University of Tokyo, Japan

**Keywords:** X-ray multilayer mirrors, synchrotron applications, X-ray monochromators

## Abstract

The results of a study of the structural and reflective characteristics of short-period multilayer X-ray mirrors based on Mo/B_4_C at wavelengths 1.54 Å, 9.89 Å and 17.59 Å are presented. The period of the samples varied in the range 8–35 Å. The average widths of the interfaces were ∼3.5 and 2.2 Å at one and the other boundaries, with a tendency for weak growth with any decrease in the period. The interlayer roughness was ∼1 Å. The research results indicate promising prospects for the use of multilayer Mo/B_4_C mirrors for synchrotron applications.

## Abstract 2

Short-period Mo/B_4_C multilayer mirrors are shown to be extremely promising for use in synchrotron applications.

## 1. Introduction

X-ray multilayer mirrors (MLMs) are used in various fields of science and technology, such as solar astronomy, X-ray lithography, X-ray microscopy and spectroscopy (Martínez-Galarce *et al.*, 2013 ▸; Chkhalo & Salashchenko, 2013 ▸; Akhsakhalyan *et al.*, 2017 ▸; Fogelqvist *et al.*, 2017 ▸). One of the important practical applications of X-ray multilayer mirrors is monochromatization and nanofocusing systems at synchrotron radiation stations (Rashchenko *et al.*, 2020 ▸; Morawe, 2019 ▸; Morawe *et al.*, 2017 ▸; Sawhney *et al.*, 2011 ▸; Chkhalo, Garakhin, Malyshev *et al.*, 2022 ▸; Mimura *et al.*, 2010 ▸; Yumoto *et al.*, 2022 ▸; Koyama *et al.*, 2022 ▸). The possibility of enabling the mirror to intercept as much of the synchrotron radiation beam as possible is one of the aspects that must be taken into account when developing reflective elements for such applications. To solve this problem it is necessary to increase the size of the mirror or the range of operating angles, which, in accordance with the Bragg condition, leads to the need to reduce the period of the MLM.

Another aspect of the use of multilayer mirrors is related to their spectral selectivity. Beams with a monochromatization level of 0.3–1% are sufficient in a number of X-ray diffraction problems (Leake *et al.*, 2019 ▸). In this case, the use of MLMs increases the intensity of the probe beam by two orders of magnitude compared with single crystals.

The third aspect is related to the increase in photon energy available at the latest generation of synchrotrons. To maintain the efficiency of collecting radiation from the source, the wavelength shortening automatically requires a reduction in the periods of the MLM.

All these aspects make it relevant to undertake new and additional studies of the currently known types of multilayer mirrors with short and ultrashort periods.

The most versatile and widely used short-period MLMs are mirrors based on a pair of W/B_4_C materials (Walton, 1997 ▸; Windt *et al.*, 2002 ▸; Pradhan *et al.*, 2018 ▸; Bibishkin *et al.*, 2005 ▸). The disadvantages of these mirrors are the insufficiently high (∼1%) spectral selectivity and a sharp increase in interlayer roughness due to the loss of W film continuity at periods shorter than 1.3 nm (Vainer *et al.*, 2006 ▸). Therefore, the search for alternative pairs of materials for the synthesis of short-period structures is extremely important. Theoretical calculations show that a promising pair of materials for this problem is Mo/B_4_C.

The reflective characteristics of Mo/B_4_C X-ray multilayer mirrors in the soft X-ray wavelength range have been studied and data on the thermal stability of structures at temperatures up to 600°C are given (Barthelmess & Bajt, 2011*a* ▸,*b* ▸; Zhu *et al.*, 2020 ▸; Choueikani *et al.*, 2013 ▸; Niibe *et al.*, 2002 ▸; Jankowski & Perry, 1991 ▸). In synchrotron studies, Mo/B_4_C MLMs have been used for X-ray monochromatization in the photon energy range 6–15 keV (Liu *et al.*, 2009 ▸). However, due to their rather large period, of more than 3 nm, the spectral selectivity of the mirrors was worse than 1%.

It is possible to increase the spectral selectivity of an MLM by shortening the period. However, Mo/B_4_C MLMs with ultrashort periods have not yet been sufficiently studied. In particular, the work of Andreev *et al.* (2003 ▸) should be noted, where the reflective characteristics of Mo/B_4_C MLMs with ultrashort periods of 1.22–2.09 nm in the soft X-ray range were studied. At a period of *d* = 1.22 nm at a wavelength of 0.834 nm, a reflection coefficient of 1.95% was obtained. However, the reflection coefficients in the hard X-ray range have not been studied. Neither have studies previously been undertaken of the internal structure, knowledge of which would make it possible to predict the X-ray optical characteristics of such mirrors in a wide range of photon energies.

This article presents the results of a systematic study of the reflective and structural characteristics of Mo/B_4_C multilayer mirrors with periods from 35 to 8 Å. Using laboratory and synchrotron X-ray reflectometry of specular reflection and diffuse scattering, the electron density profiles of materials in these MLMs, their interface widths and interlayer roughness were determined. The data obtained will make it possible to reliably predict their X-ray reflection coefficients across a wide range of photon energies.

## 2. Experimental methods

Synthesis of the multilayer Mo/B_4_C structures was carried out by magnetron sputtering on the facility described by Zabrodin *et al.* (2013 ▸). Before the technological process began, the pressure of the residual gases in the chamber was at a level of 10^−7^ mbar. High-purity (99.998%) argon was used as the working gas with a pressure of 9 × 10^−4^ mbar. The magnetrons were powered by sources of stabilized current of our own design. During deposition, the voltages were 296 V for Mo and 267 V for B_4_C, the currents were 200 mA for Mo and 1200 mA for B_4_C. Accordingly, the film growth rates were 0.18 nm s^−1^ for Mo and 0.56 nm s^−1^ for B_4_C. The Mo/B_4_C multilayer mirrors were sputtered on round Si substrates with a diameter of 100 mm and effective roughness of 3 Å in the spatial frequency range 0.025–64 µm^−1^.

The dependences of the reflection and scattering coefficients on the grazing angle of radiation at a wavelength of 1.54 Å were measured using a laboratory high-resolution four-crystal diffractometer PANalitycal X’Pert Pro. Similar measurements were carried out at the PHASE station of the Kurchatov specialized source of synchrotron radiation KISI-Kurchatov (Chernyshov *et al.*, 2009 ▸; Senin *et al.*, 2017 ▸). The operating radiation energy range of this station is 3.5–50 keV. The station is equipped with a two-crystal monochromator providing an energy resolution of Δ*E*/*E* = 5 × 10^−4^.

Measurements at wavelengths of 17.59 Å (Fe *L*α) and 9.89 Å (Mg *K*α) were carried out on another laboratory reflectometer with a grating grazing-incidence monochromator spectrometer. More details about the reflectometer are given by Bibishkin *et al.* (2004 ▸).

The structural parameters of the samples (period, layer thicknesses, interlayer roughness, electron density profile) were determined by simultaneously fitting the reflection curves at wavelengths of 1.54, 9.89 and 17.59 Å using the *Multifitting* program (Svechnikov *et al.*, 2017 ▸; Svechnikov, 2020 ▸).

## 3. Experimental results obtained using laboratory methods

In the framework of the experiments, nine Mo/B_4_C MLMs were synthesized with periods ranging from 8 to 35 Å. The number of layers in each mirror was chosen so that the total thickness of all samples remained unchanged. The proportion of molybdenum in the period was 0.45–0.5.

**Figure 1:**
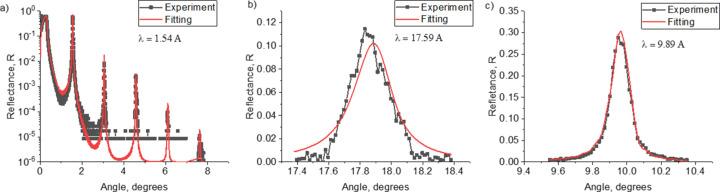
Experimentally measured dependences of the reflectance (black curves) and fitting of experimental data (red curves) for the RS-154 sample at wavelengths λ = 1.54 Å (*a*), 17.59 Å (*b*) and 9.89 Å (*c*).

**Figure 2:**
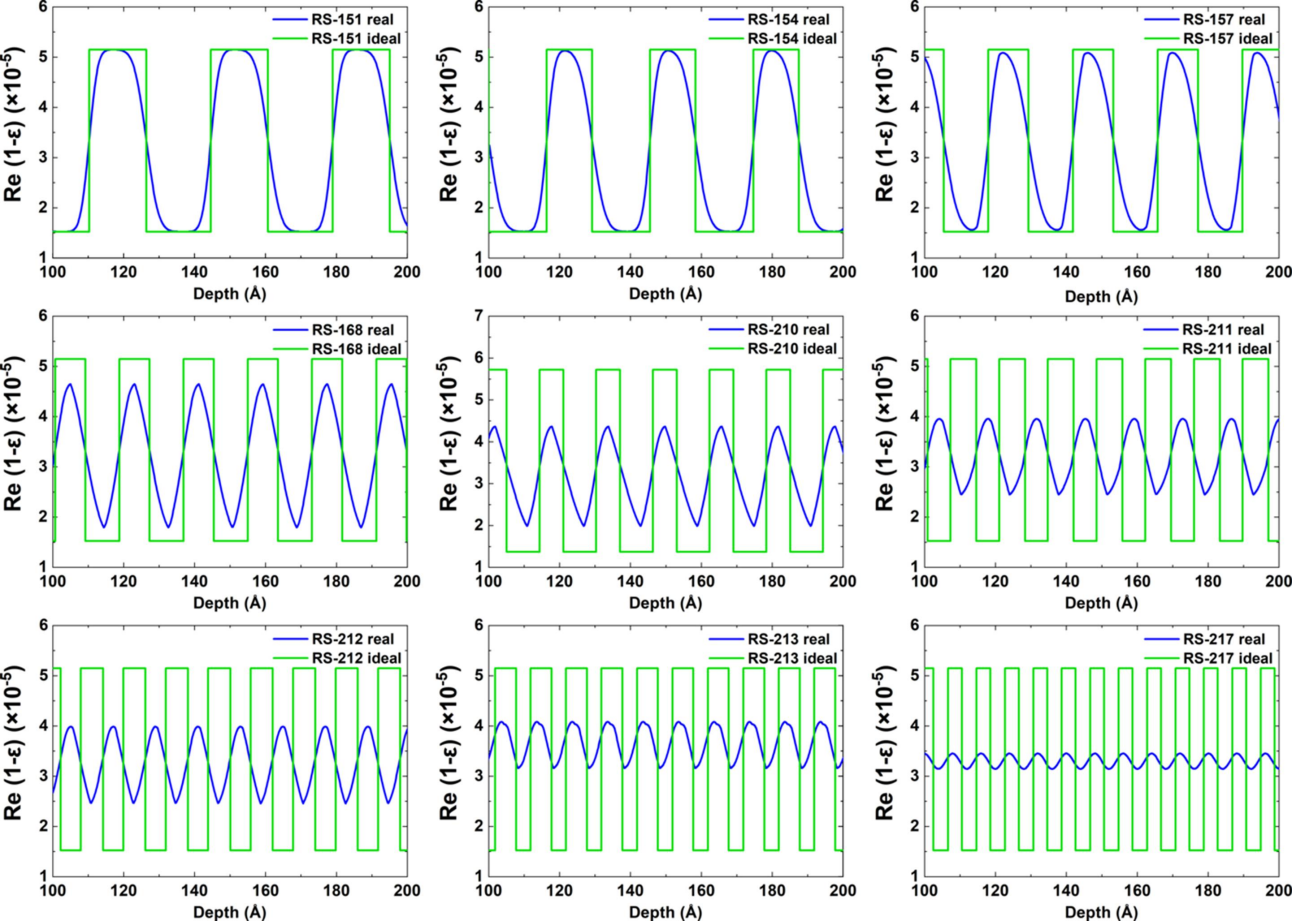
Electron density profiles of samples RS-151 to RS-217 reconstructed from X-ray reflection data. The blue curve corresponds to the electron density distribution deep into the structure with allowance for the interfaces; the green curve corresponds to the case of an ideal structure with zero interfaces.

**Figure 3:**
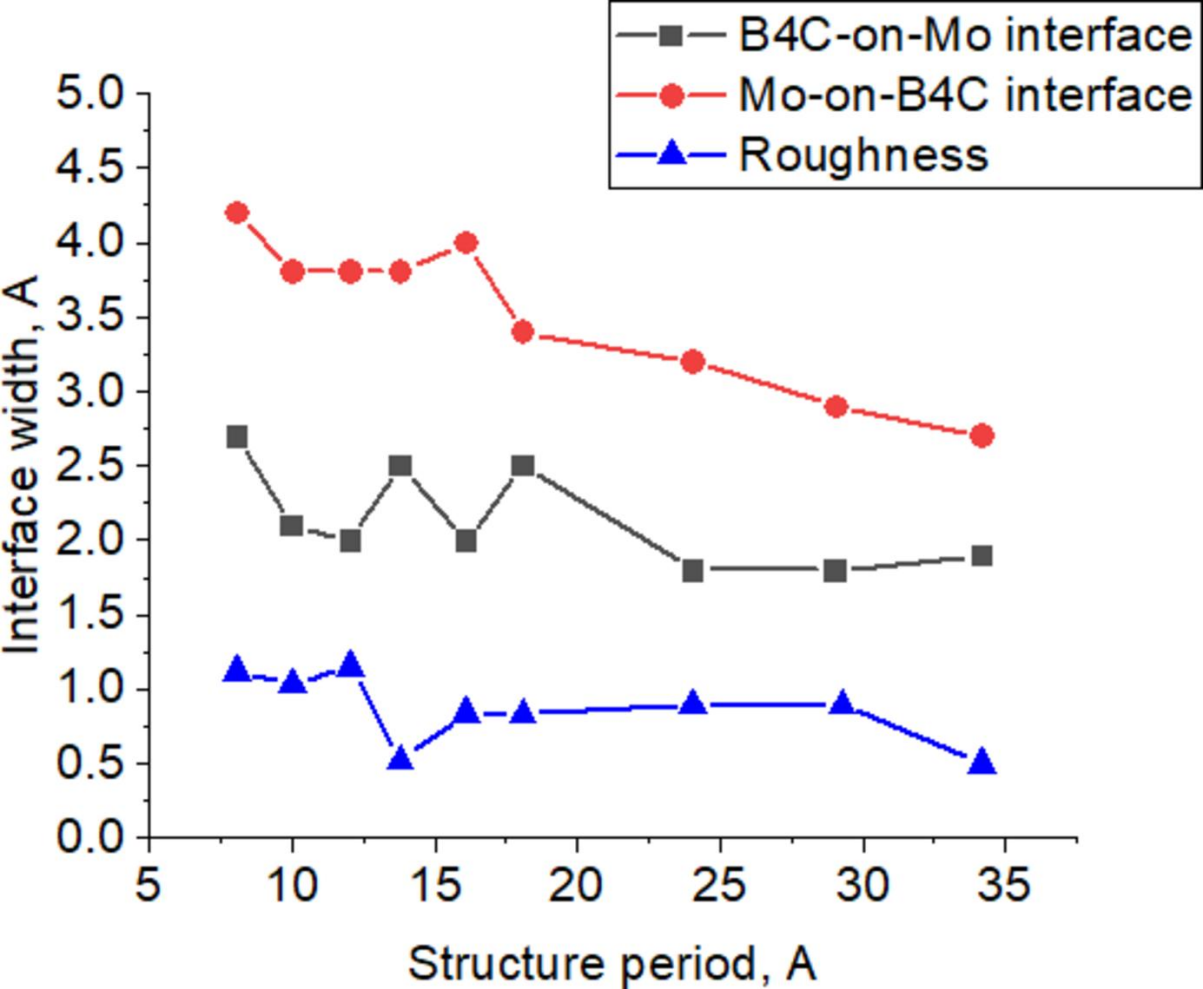
The dependence of the Mo-on-B_4_C (red curve with circles) interface width, B_4_C-on-Mo (black curve with squares) interface width and the interlayer roughness (blue curve with triangles) on the period of the structure.

At the first stage, the angular dependences of the reflection coefficient at wavelengths of 1.54, 9.89 and 17.59 Å were measured and the experimental data were fitted for all samples. In Fig. 1 ▸ the black curves with dots show the experimental data and the red curves show the results of fitting for the RS-154 sample. The structural parameters determined in this way for all samples are shown in Table 1 ▸. The electron density profiles are shown in Fig. 2 ▸. The dependence of the interface width on the period of the structure is shown in Fig. 3 ▸ (two upper curves). It should be noted that reflectometry does not allow one to unambiguously determine which interface, in our case Mo-on-B_4_C or B_4_C-on-Mo, corresponds to the values obtained as a result of fitting. However, based on Svechnikov *et al.* (2018 ▸), where the effect of various interlayers, including B_4_C, on the Mo/Be interfaces of MLMs was studied, it can be assumed that the Mo-on-B_4_C interface is more extended.

Interface formation is affected by several mechanisms, the main of which are growth roughness and mutual mixing of materials, primarily due to the ballistic effect of the introduction of atoms with high energies (a few to tens of eV) of the condensate entering the film, as well as diffusion enhanced by chemical interaction. It is possible to influence one or other of these mechanism to affect interface formation by varying the parameters of the technological process involved in the growth of the MLM. Therefore, it is important to separate the contributions of growth roughness and of material mixing to the total interface width in order to optimize the MLM growth processes. Thus, it makes sense to apply methods of ion polishing of the layers (Spiller, 1989 ▸) in the case of a predominance of roughness in the structure. Correspondingly, in the case of a prevalence of diffusion processes, there is an effective way of reducing this by using barrier layers and optimizing the energy of the atoms of condensate entering the substrate (Polkovnikov *et al.*, 2022 ▸).

**Figure 4:**
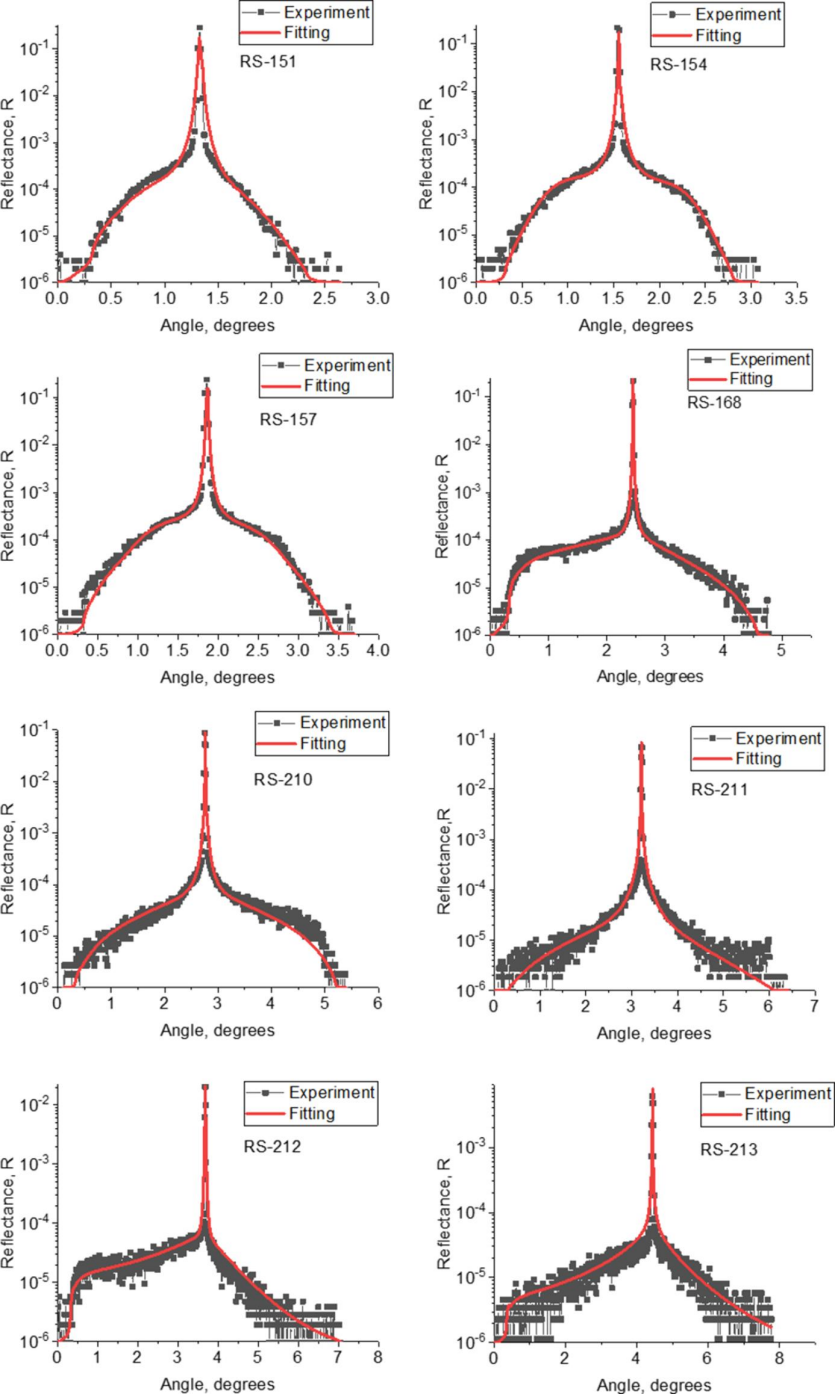
Experimental (black) and fitted (red) diffuse scattering curves for RS-151 to RS-213 structures.

In order to separate the contributions of the above effects, the method of measuring the diffuse X-ray scattering curves was used, because the interlayer roughness is responsible for scattering, while mixing leads to only a slight decrease in the intensity of the scattered radiation due to a decrease in the electron density jump at the boundaries. Within the framework of this approach, after measuring the specular reflection curves at a wavelength of λ = 1.54 Å, we measured the rocking curves obtained as follows: the sample and the detector were rotated relative to a stationary radiation source by an angle corresponding to the first Bragg peak, after which the reflection coefficient was measured at various sample rotation angles. The detector position remained fixed in this case. This type of measurement makes it possible to detect radiation scattered over a wide range of angles, since, for all angles of radiation incidence on the sample, the condition of constructive interference is satisfied: sinϑ_in_ + sinϑ_sc_ ≃ 2sinϑ_Br_, where ϑ_in_ is the grazing angle of incidence on the sample, ϑ_sc_ is the angle between the scattered beam and sample plane, and ϑ_Br_ is the Bragg angle. The measured diffuse scattering curves and their fittings for all samples are shown in Fig. 4 ▸.

A linear growth model was chosen (Asadchikov *et al.*, 2001 ▸; Bass, 1995 ▸) to calculate the roughness of the boundaries, where the power spectral density (PSD) function of the interfaces is partially inherited from the previous ones, and partially replaced by a growth model (Stearns, 1993 ▸),

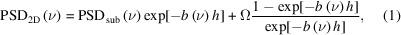

where PSD_sub_(ν) corresponds to the substrate, exp[−*b*(ν)*h*] is the inheritance factor, Ω is the volume of the deposited particle (atom, molecule or cluster), *h* is the film thickness and *b*(ν) is the function of surface relaxation, which is represented as a polynomial in powers of spatial frequency: *b*(ν) = 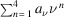 . The ABC model (Bass, 1995 ▸) was chosen as the model describing the PSD function of the substrate. A PSD function of a substrate in this case is described by the formula

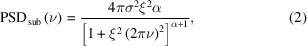

where σ is the total RMS substrate roughness height, α is the fractal dimension, determining the rate at which the spectrum decays into the high-frequency region, and ξ is the cross (transverse, along the layer) correlation length. Longitudinal correlation (in the direction perpendicular to the layer) *L* was calculated from the function of surface relaxation: *L* = *b*(ν)^−1^.

The value of the roughness of layers was obtained by integrating the PSD function in a given range of spatial frequencies, which was determined from the condition 2πν = *k*(cosϑ_0_ − cosϑ), where ν is the spatial frequency value, ϑ_0_ is the angle corresponding to the specular reflection, ϑ is the scattering angle and *k* is the wavevector. The value of the scattering angle close to the Bragg peak was chosen to obtain the lower boundary of integration while the value corresponding to the angle of maximum scattering was chosen to obtain the upper boundary. Table 2 ▸ shows statistical properties of the roughness of Mo/B_4_C MLMs reconstructed from diffuse scattering data.

In Fig. 3 ▸ the curve marked with triangles shows the dependence of the roughness on the MLM period, obtained with allowance for the analysis of diffuse scattering and specular reflection. Despite a noticeable scatter of experimental data from sample to sample, most likely caused by errors in solving the inverse problem, nevertheless, certain conclusions can be drawn about the structural parameters and their changes with a decrease in the period of the Mo/B_4_C MLM. It can be argued that the average interface width at the Mo-on-B_4_C boundary was ∼3.5 Å and at the B_4_C-on-Mo boundary was ∼2.2 Å. The interlayer roughness was ∼1 Å. A weak growth of interfaces widths and interlayer roughness with the period decrease is observed. The length of the transverse correlation of roughness is determined by the characteristics of the substrate and is within 10 µm. The value of the longitudinal correlation length is at a level of tens of nanometres.

Another factor affecting the deterioration of the reflective characteristics of multilayer mirrors, especially short-period ones, is the period drift with growth. In our case it was about 1%. The last two columns of Table 1 ▸ show the calculated values of the reflection coefficient and spectral selectivity corresponding to a case without any change in the period. As can be seen from the table, the period drift strongly limits the experimental reflection coefficients and spectral selectivity. At a wavelength of 1.54 Å, Mo/B_4_C MLMs provide a reflectance of 50% and a spectral selectivity of 0.4% at a period of 18 Å with zero period drift. Compared with W/B_4_C mirrors with similar reflection coefficients, the spectral selectivity of Mo/B_4_C MLMs is almost two times higher. The reflection coefficients also increase with increases in photon energy.

**Figure 5:**
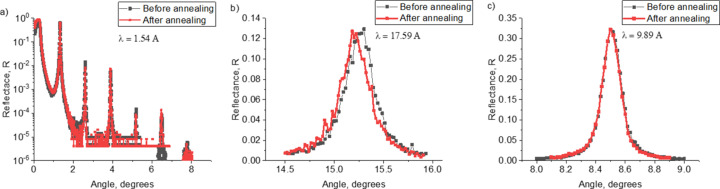
Angular dependences of the Mo/B_4_C reflectance of the RS-151 structure before annealing (black curves with dots) and after annealing (red curves) for 1 h at a temperature of 300°C at wavelengths of 1.54 Å (*a*), 17.59 Å (*b*) and 9.89 Å (*c*).

Within the framework of the performed studies, the effect of vacuum annealing on the reflective characteristics of the synthesized Mo/B_4_C structures was also investigated. The mirrors were annealed for an hour at temperatures from 150 to 300°C. The measurements of the reflection coefficient dependences on the angle of incidence of the radiation before and after annealing showed no degradation of the reflective characteristics of the structures under thermal exposure, which indicates their thermal stability. An example of such an analysis for sample RS-151 is shown in Fig. 5 ▸.

## 4. Experimental results obtained using synchrotron radiation

**Figure 6:**
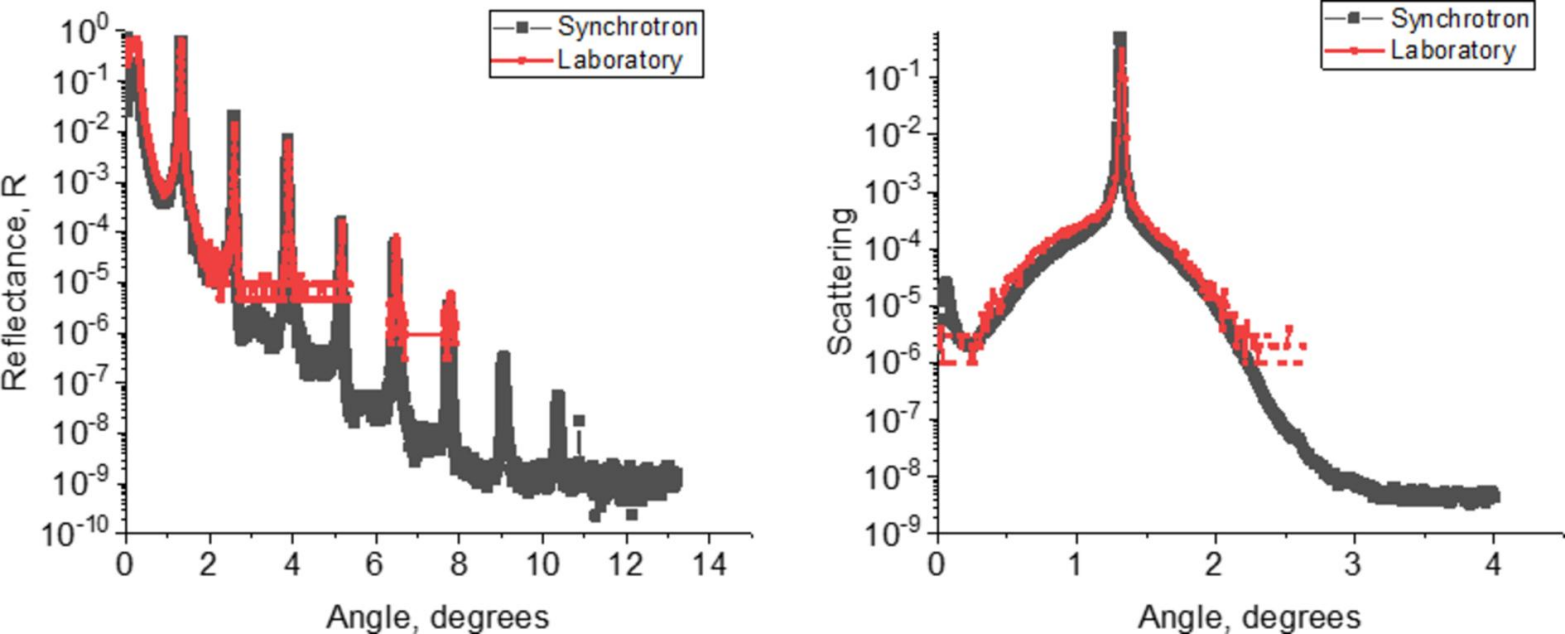
Experimental measurements of the angular dependence of the reflection coefficient (*a*) and diffuse scattering (*b*) carried out in the framework of laboratory (red curve) and synchrotron (black curve with dots) studies.

Due to the larger dynamic range of X-ray intensity at the synchrotron and in order to refine the results obtained by laboratory methods, several samples were studied at the KISI synchrotron. Fig. 6 ▸ shows a comparison of the experimental curves of the angular dependence of the reflection coefficient (*a*) and diffuse scattering (*b*) obtained in the framework of laboratory and synchrotron measurements for the RS-151 sample.

**Figure 7:**
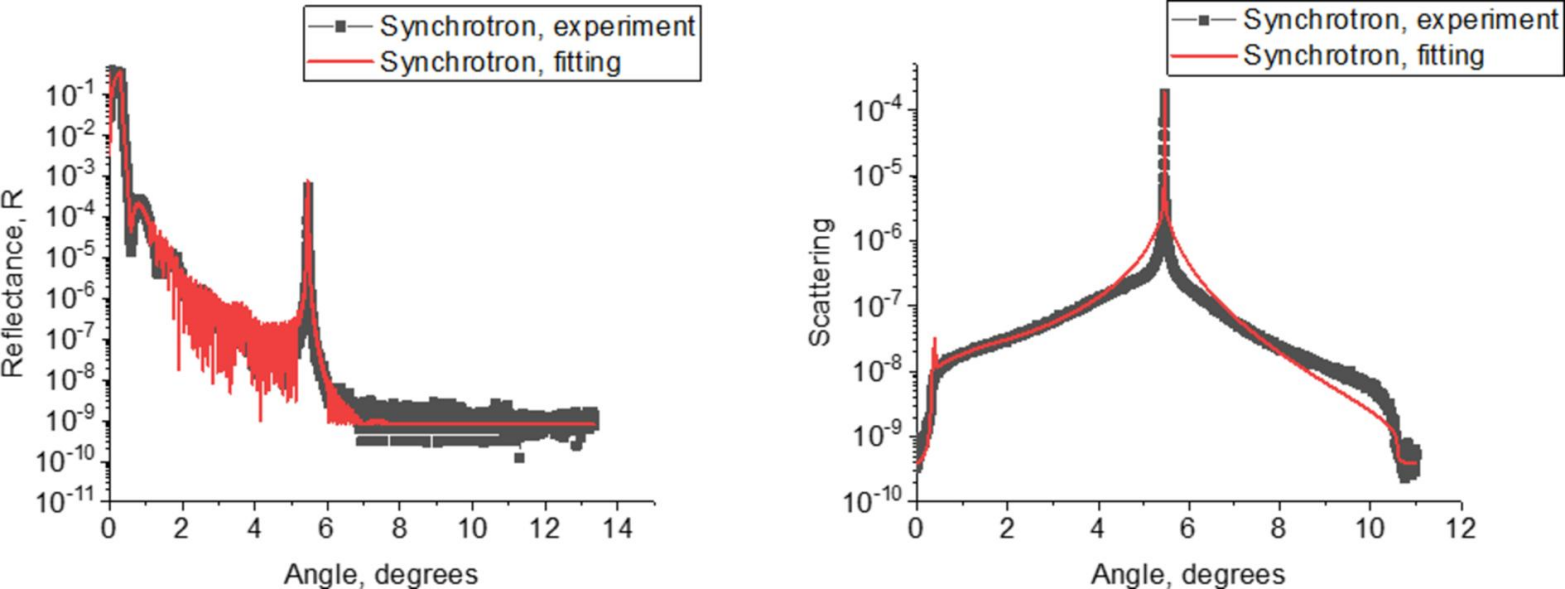
Experimental measurements of the angular dependence of the reflection coefficient (*a*) and diffuse scattering (*b*) carried out in the framework of synchrotron studies. Black curves with squares correspond to experimental data, red curves to their fitting.

From the presented data we can conclude that the synchrotron and the laboratory measurements are in good agreement, which confirms their reliability. Fig. 7 ▸ shows the results of measuring the reflection coefficient (*a*) and the diffuse scattering (*b*) for the RS-217 sample, carried out at the synchrotron, as well as the fitting of the experimental data.

A slight discrepancy in experimental and fitting curves of diffuse scattering could be explained by the fact that this fitting refers to the sample with extremely short period of 8 Å. It is possible that, for this structure, a significant contribution to scattering is made by spatial inhomogeneities (individual clusters and crystallites), which are not taken into account by our model, which considers only scattering from interfaces. These effects were observed in multilayer W/Si mirrors, which was considered by Chkhalo, Garakhin, Kumar *et al.* (2022 ▸).

## 5. Conclusion

Within the framework of the study the reflective and structural characteristics of Mo/B_4_C multilayer mirrors with periods from 8 to 35 Å were studied. The main results of the study were as follows.

First, the value of the interlayer roughness remains practically unchanged for all structures σ ≃ 1 Å with a slight tendency to increase at period values of about 10 Å. Such behaviour of the interlayer roughness indicates preservation of the continuity of such ultrathin films and was observed earlier in Cr/Be MLM (Pleshkov *et al.*, 2021 ▸). In that work, this effect was explained by the formation of chromium beryllides in the case of ultrathin layers. Apparently, molybdenum boride films are formed in our case as well.

Second, the main contribution to the interface widths is made by mixing of the film materials at the boundaries. At the same time, the Mo-on-B_4_C border is the most blurred. The widths of the interfaces varied from 3 to 4 Å with the period decrease from 35 to 8 Å.

Thirdly, the length of the transverse correlation of roughness is determined by the characteristics of the substrate and is within 10 µm. The value of the longitudinal correlation length is at a level of tens of nanometres.

Fourth, the reflection coefficients and the spectral selectivity of the Mo/B_4_C MLMs studied in this work are limited by the period drift over the depth of the samples. In our case the drift value is at a level of 1%. In cases of an absence of period drift, the reflection coefficient at a wavelength 1.54 Å is about 50% and a spectral selectivity of 0.4% is achieved at a period of 18 Å. In general, the Mo/B_4_C MLMs significantly outperform W/B_4_C mirrors in terms of spectral selectivity with comparable reflectance. Furthermore, there is no degradation of the interfaces in the Mo/B_4_C structures at periods up to 10 Å.

Fifth, the study of thermal stability during vacuum annealing up to 300°C showed the high stability of the reflective characteristics of short-period Mo/B_4_C mirrors as had been previously observed for samples with long periods (Shaposhnikov *et al.*, 2023 ▸).

Sixth, a comparison of the results of measurements carried out on laboratory equipment shows good agreement with measurements carried out at the KISI synchrotron. This fact indicates the adequacy of the laboratory methods we have developed for studying the reflective characteristics and internal structures, including those for multilayer mirrors with ultrashort periods. Thus, on the basis of the study performed, it can be concluded that short-period Mo/B_4_C multilayer mirrors are extremely promising for use in synchrotron applications.

## Figures and Tables

**Table 1:** Structural parameters of the synthesized Mo/B_4_C structures *d* is the period of the structure in Å, *R* is the reflection coefficient in the first Bragg peak at a wavelength of 1.54 Å in %, *N* is the number of periods, σ_Mo_ is the width of the B_4_C-on-Mo interface in Å, σ_B4C_ is the width of the Mo-on-B_4_C interface in Å, Δλ/λ is the spectral selectivity in the first Bragg peak in %, R* and Δλ/λ* are the calculated reflection coefficient and spectral selectivity in % in the first Bragg peak with zero period drift.

Sample	*d*, Å	*R*, %	*N*	σ_B4C_, Å	σ_Mo_, Å	Δλ/λ, %	R*, %	Δλ/λ*, %
RS-151	34.16	63.3	115	2.7	1.9	3	66.4	2
RS-154	29.26	57.8	135	2.9	1.8	1.7	62	1.5
RS-157	24.05	44.5	162	3.2	1.8	1.6	57.8	0.9
RS-168	18.08	40	220	3.4	2.5	0.7	50	0.4
RS-210	16.1	19.5	250	4	2	1	44.8	0.23
RS-211	13.79	10.1	285	3.8	2.5	0.9	21.4	0.15
RS-212	12.05	4	335	3.8	2	0.8	27.6	0.12
RS-213	10	0.5	400	3.8	2.1	0.5	11	0.1
RS-217	8.05	0.1	500	4.2	2.7	0.4	1.5	0.069

**Table 2:** Statistical properties of the roughness of Mo/B_4_C MLMs reconstructed from diffuse scattering data

Sample	Period	Roughness, Å	Longitudinal correlation, nm (at a frequency of 3 µm^−1^)	Cross correlation, µm
RS-151	34.16	0.5	38	9
RS-154	29.04	0.9	142.6	9
RS-157	24.05	0.89	76.6	10
RS-168	18.08	0.83	32.1	9
RS-210	16.1	0.84	37.3	9
RS-211	13.79	0.52	83.6	10
RS-212	12.05	1.15	53.6	10
RS-213	9.98	1.03	49.3	9
RS-217	8.05	1.12	20.1	10
